# X-ray tomographic data of planktonic foraminifera species *Globigerina bulloides* from the Eastern Tropical Atlantic across Termination II

**DOI:** 10.46471/gigabyte.5

**Published:** 2020-09-25

**Authors:** Stergios D. Zarkogiannis, Vincent Fernandez, Mervyn Greaves, P. Graham Mortyn, George Kontakiotis, Assimina Antonarakou

**Affiliations:** ^1^ Faculty of Geology & Geoenvironment, Department of Historical Geology-Paleontology, School of Earth Sciences, National & Kapodistrian University of Athens, Greece; ^2^ Imaging and Analysis Centre, Natural History Museum, London, UK; ^3^ Department of Earth Sciences, University of Cambridge, Cambridge, UK; ^4^ Institute of Environmental Science and Technology (ICTA), Universitat Autònoma de Barcelona, Spain; ^5^ Department of Geography, Universitat Autònoma de Barcelona, Spain

## Abstract

Increased planktonic foraminifera shell weights were recorded during the course of Termination II at a tropical site off the shore of the Mauritanian coast. In order to investigate these increased shell mass values, a series of physicochemical analyses were performed, including X-ray computed tomography (CT). The data are given here. Furthermore, the relevant CT setup, scanning, reconstruction, and visualization methods are explained and the acquired datasets are given, together with 3D volumes and models of the scanned specimens.

## Introduction

Planktonic foraminifera are unicellular, marine microorganisms (protists) that produce calcium carbonate (CaCO_3_) shells (tests). Their biomineralization plays a major role in controlling the alkalinity and carbonate chemistry of the photic zone of the world ocean and is intimately related to atmospheric *p*CO_2_ and the regional or global budgets of the carbon system [1]. Planktonic foraminifera are known to considerably alter their shell weight throughout the paleoceanographic record [2] and the degree of this alteration is a function of latitude [3]. In an accompanying study of a tropical Atlantic core [4] we report relatively small variation in the shell weights of the planktonic species *Globigerina bulloides* (NCBI: txid69025; urn:lsid:marinespecies.org:taxname:113434) during the last two climatic cycles, with the exception of an increased weight event during Termination II. In order to identify the cause of these elevated shell mass values prior to their dissolution for geochemical analyses, specimens from samples that surround this event were tomographically analyzed to gain insights about the microscopic fossils. Analyses showed that the observed increase in the recorded shell weights is the result of both thicker and thus more massive or heavier tests, but also partly an artifact caused by increased sediment contamination.

Laboratory X-ray micro-computed tomography (μCT) is a fast-growing method in various scientific research applications, including micropaleontology, that allows non-destructive imaging of fossils [5]. μCT analysis can give valuable information about seawater carbonate chemistry [6], the preservation state of fossil foraminifera specimens [7, 8], changes in the morphology [9] and the thickness of foraminifera shells [10–12], while its combination with shell geochemical measurements is a powerful tool in the study of foraminifera shell biomineralization [13, 14]. Despite the multiple uses of CT microscopy in foraminifera or paleoceanographic studies, and its potential to archive specimens that are frequently destroyed for geochemical analyses, only limited raw data are available for reanalysis.

The present dataset consists of tropical foraminifera samples that record a weight anomaly during the penultimate deglaciation period. The direct impact of foraminifera shell-building intensities on the alkalinity of the ocean and its atmospheric CO_2_ exchanges stresses the importance to closely examine their shell mass and keep a record until their role is better understood. Because weighing and CT scanning are non-destructive analytical techniques, they are ideal to precede any destructive paleoceanographic analysis. CT analysis focused mainly on the biometric characteristics of the specimens that underwent geochemical analysis. However, although the specimens have been dissolved, their virtual models, together with the original acquisition data, are provided here and readily available for future use.

## Implementation

**Figure 1.**
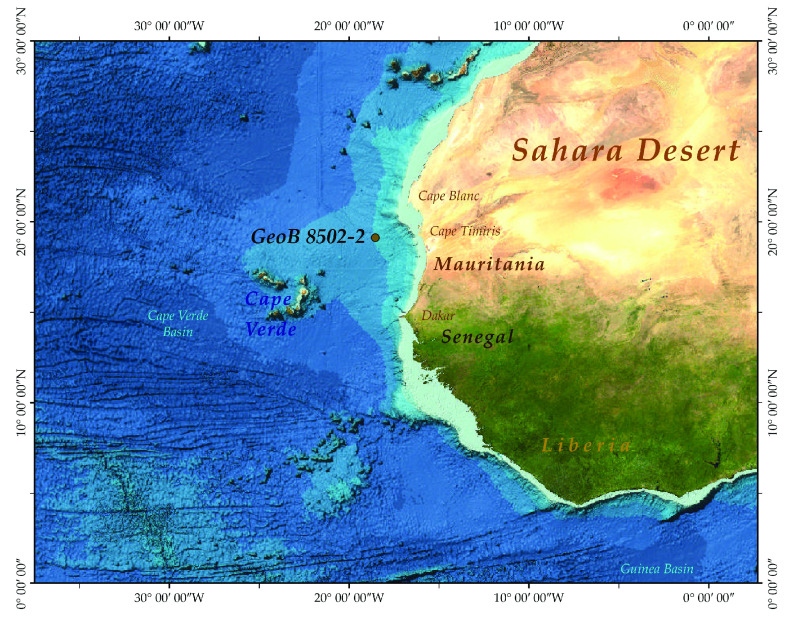
Location of the sampling site of the studied core GeoB 8502-2 along the north-western African margin, located approximately 250 km off the shore of the Mauritanian coast *and* viewed in OpenStreetMap.

**Figure 2.**
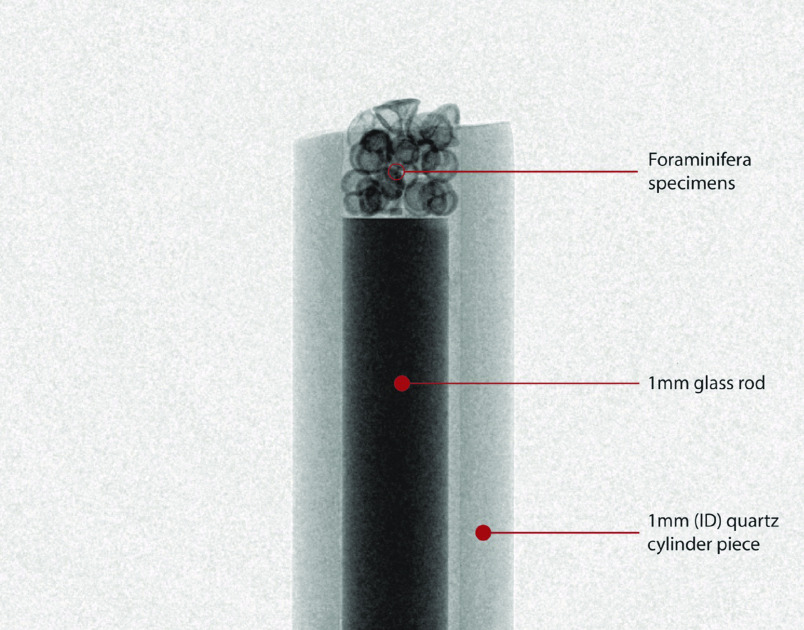
Customized sample container for foraminifera X-ray computer tomography analysis.

The specimens used in the present analysis are from core GeoB850-2 (19^°^ 13.27’ N, 18^°^ 56.04’ W), which was retrieved from a depth of 2,956 m approximately 250 km off the shore of the Mauritanian coast (Figure 1). The core samples were initially wet-sieved, and the coarse fraction (<63 μm) was subsequently separated in different sieve fractions. The analyzed specimens were picked from the 300–355 μm size fraction, and, after scanning, were dissolved for geochemical analyses. Samples from GeoB cores can be obtained from MARUM GeoB Core Repository. Additional information about the study area, the materials and the methods used for the present analyses can be found in the accompanying paper [4]. Nano-CT scanning was conducted with a Zeiss Versa 520 3D X-ray microscope at the X-ray Microscopy (XRM) facility of the Natural History Museum, London. Samples were analyzed in batches of nine shells using a customized sample container. The container was made using a crystal clear quartz capillary cylinder piece of 2 cm height and 1 mm inner diameter, blocked at one end with a 1 mm glass rod. The glass rod was forced into the quartz cylinder until approximately 1 mm from its open end, creating a space of ≈0.8 mm^3^ for the microfossils (Figure 2). The cylindrical piece was stabilized around the glass rod with nail varnish and the rod was inserted in the sample mounting pin vise. The foraminifera specimens were subsequently transferred into the container with a saddle brush, together with a calcite microcrystal as standard, and were stabilized with a drop of tragacanth gum. The samples were left to dry before being loaded into the scanner for analysis.

The specimens were picked from the 300–355 μm size fraction and were analyzed at a magnification of 4× by acquiring 1601 projections. Sample mount, X-ray source and detector geometry were kept constant throughout the scans. For optimum foraminifera shell acquisition, the voltage was set to 100 kV, the current at 90 μA (providing an overall power of 9 W), and the exposure time was set to 2 s. A scan resolution voxel size of ∼1.2 μ*m*^3^ was typically achieved using this set up. The exact voxel of each scan, as provided by the instrument, is given in the text file that accompanies each data set, and it is the one used for segmentation.

Image segmentation followed by a surface determination function was performed using Avizo^®^ software without applying a de-noising filter. The watershed algorithm was mainly used, which automatically separates adjacent areas on a grayscale image into different ‘materials’, based on user-defined attributes of certain pixels. The segmented areas involve the shell area, the sediment contaminated area (dirt area) and the total internal void area (protoplasm area). Initially, the test, the dirt and the background areas were defined, whereas background marked were the pixels that did not refer to the two previous attributes (shell, dirt). Subsequently the background ‘material’ was deleted and the internal void area of the shell was segmented, using the ambient occlusion module, as ‘protoplasm’. The three different areas were then added together to produce the ‘cell’ area, which is the total area occupied by the foraminifer (Figure 2 in [4]).

## Availability of source data and requirements

The data presented here in detail accompany a recent paper [4], where they are summarized and discussed further. Analysis of the high-resolution CT scan images of the foraminifera specimens led to accurate determination of their cell volumes. This, combined with their weighed masses, allowed volume normalized test weights (or shell densities) to be reported for the first time. CT data are provided in the form of 16-bit TIFF image stacks, with associated voxel size and other scan settings in the accompanying text file. The virtual specimens, together with the different spatial information that resulted from the analyses of the tomographs, are given in Tables 1–3, where Shell volume is the volume of shell calcite mass, shell area is the outer surface area of the calcite mass. The ratio of shell volume/shell area (or ‘specific surface area’ [6]) is a measure of average shell thickness. Total cell volume is the sum of shell and internal void (protoplasm) volumes, and dirt % is the percentage of the volume segmented as contamination within the total cell volume.

**Figure 3.**
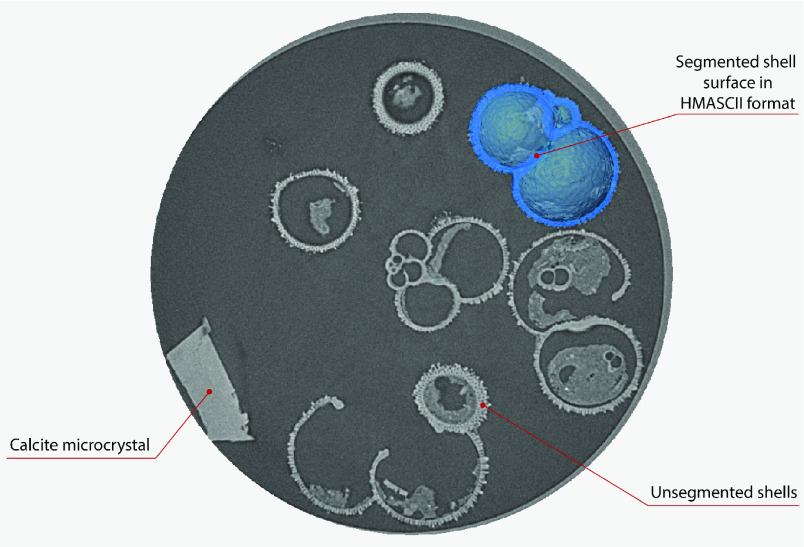
Example tomograph of scan GeoB 8502-2_Depth825, in which the HMASCII 3D volume of a processed shell is superimposed.

The processed 3D volumes of each specimen created using the surface determination function are also provided with the scan dataset in HMASCII file format. The 3D volumes can be viewed with any 3D model viewer, while the two datasets can be combined in CT image analysis software (Figure 3). Interactive views of the 3D models available in the Sketchfab repository are also embedded in the tables, enabling the virtual specimens to be interactively explored.

**Table 1:** Measurements based on the segmentation of specimens in a sample at 785 cm below the sea floor of core GeoB 8502-2, corresponding to 122.5Kyrs before present.

Sample 785 cm	Interactive 3D Models	Shell Volume (μm^3^)	Shell Area (μm^2^)	Shell thickness (μm)	Cell Volume (μm^3^)	Dirt
GeoB 8502-2_Depth785_Shell 1	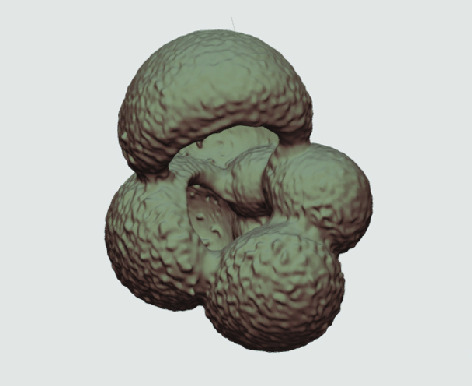	5,319,397	973,457	5.5	20,133,400	7%
GeoB 8502-2_Depth785_Shell 2	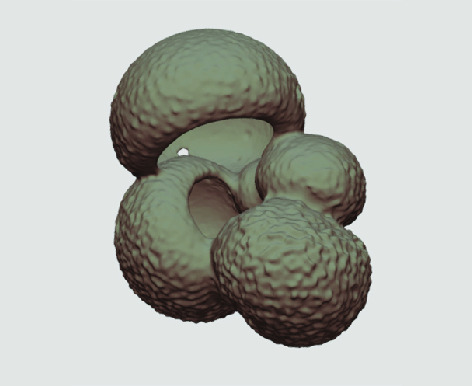	5,228,296	1,069,520	4.9	23,128,400	7%
GeoB 8502-2_Depth785_Shell 3	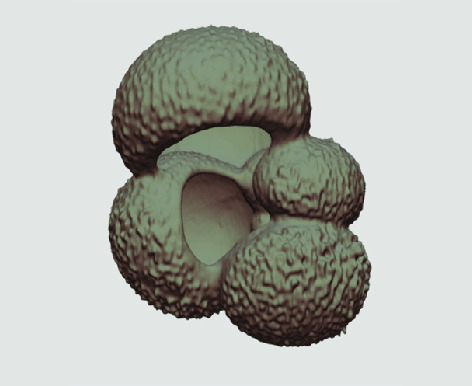	5,014,924	988,223	5.1	20,624,600	3%
GeoB 8502-2_Depth785_Shell 4	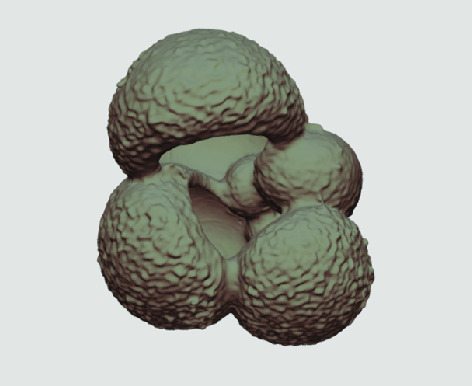	5,699,836	1,127,470	5.1	25,106,100	9%
GeoB 8502-2_Depth785_Shell 5	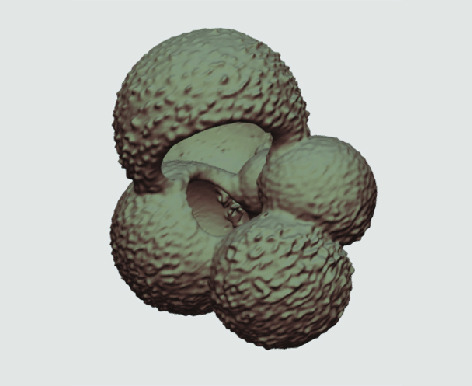	4,867,658	921,172	5.3	18,882,400	2%
GeoB 8502-2_Depth785_Shell 6	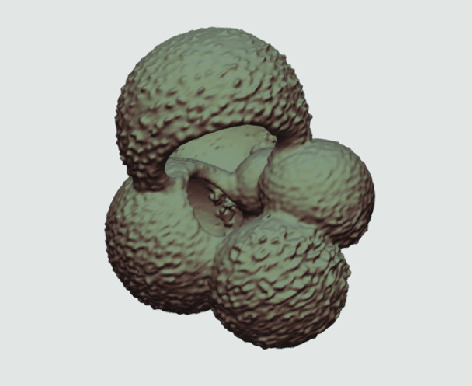	5,548,905	1,126,413	4.9	24,397,400	9%
GeoB 8502-2_Depth785_Shell 7	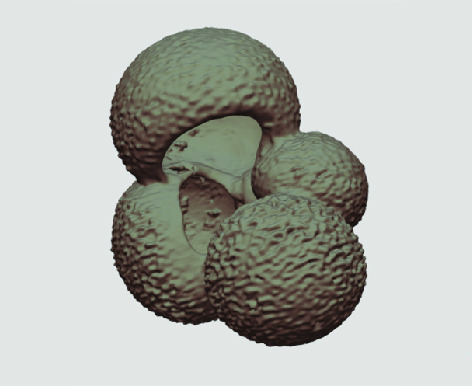	4,542,654	1,030,054	4.4	21,945,300	6%
GeoB 8502-2_Depth785_Shell 8	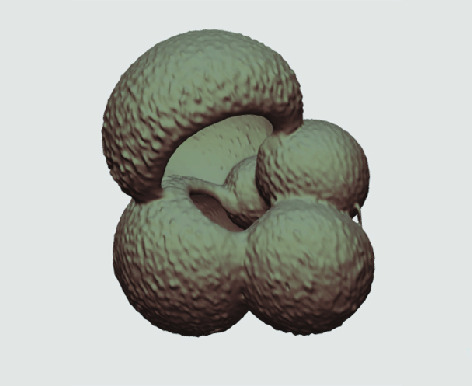	5,566,874	1,035,128	5.4	22,689,200	1%
GeoB 8502-2_Depth785_Shell 9	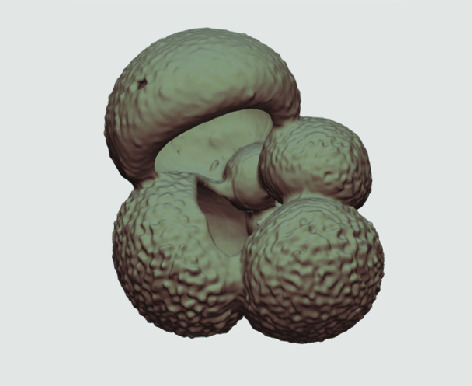	4,918,244	1,105,755	4.4	24,028,000	2%
**Average**		**5,189,643 ± 7%**	**1,041,910 ± 7%**	**5.0 ± 7%**	**22,326,089 ± 9%**	**5%**

**Table 2:** Measurements based on the segmentation of specimens in a sample at 825 cm below the sea floor of core GeoB 8502-2, corresponding to 132.2 Kyrs before present.

Sample 825 cm	Interactive 3D Models	Shell Volume (μm^3^)	Shell Area (μm^2^)	Shell thickness (μm)	Cell Volume (μm^3^)	Dirt
GeoB 8502-2_Depth825_Shell 1	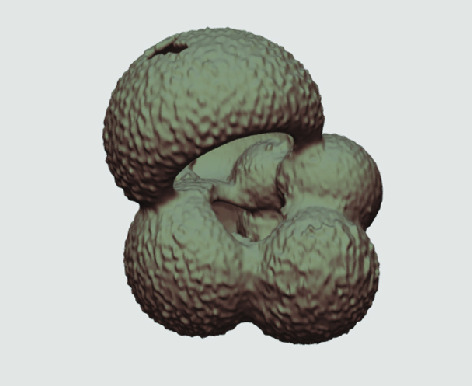	5,327,092	962,894	5.5	20,825,700	39%
GeoB 8502-2_Depth825_Shell 2	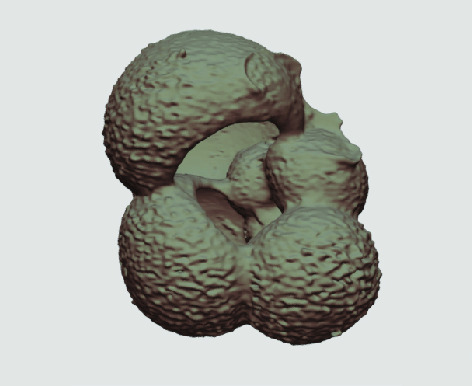	5,916,615	967,372	6.1	20,921,000	8%
GeoB 8502-2_Depth825_Shell 3	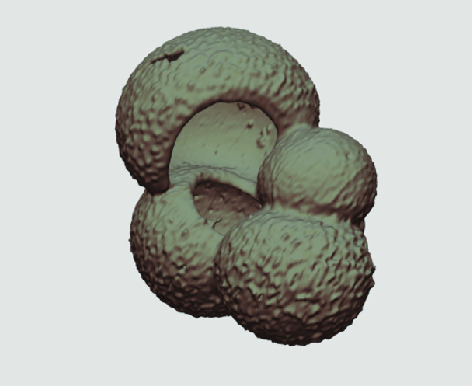	5,375,810	1,055,036	5.1	23,055,000	8%
GeoB 8502-2_Depth825_Shell 4	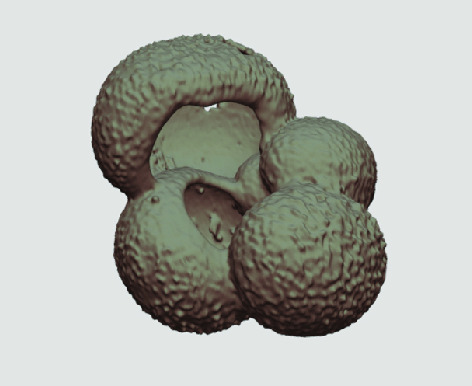	6,885,647	1,178,105	5.8	27,290,900	9%
GeoB 8502-2_Depth825_Shell 5	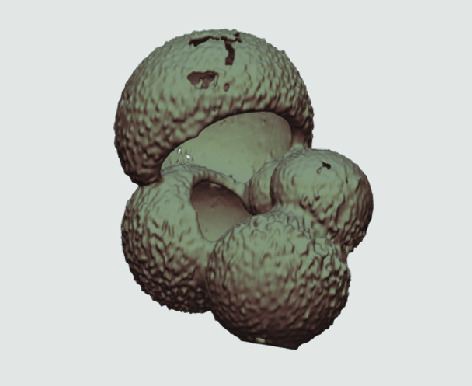	6,205,883	1,220,864	5.1	28,907,800	25%
GeoB 8502-2_Depth825_Shell 6	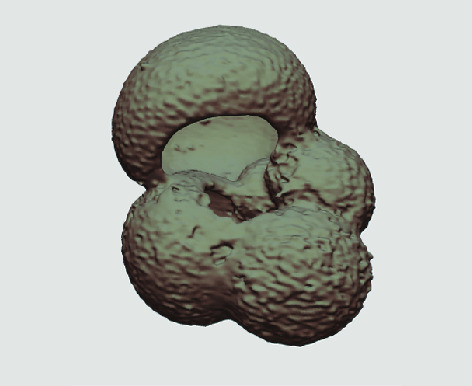	6,233,948	1,000,779	6.2	22,787,800	26%
GeoB 8502-2_Depth825_Shell 7	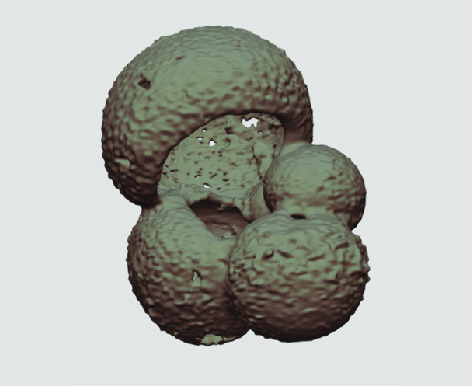	7,657,985	1,387,413	5.5	34,866,200	21%
GeoB 8502-2_Depth825_Shell 8	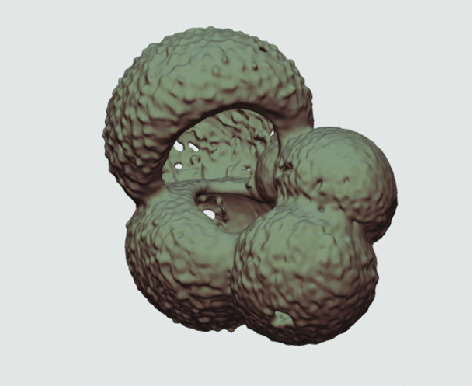	5,139,404	964,780	5.3	19,186,100	19%
GeoB 8502-2_Depth825_Shell 9	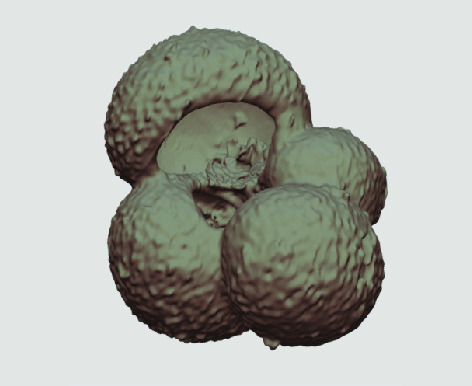	6,106,162	935,082	6.5	19,301,100	15%
**Average**		**6,094,283 ± 14%**	**1,074,703 ± 14%**	**5.7 ± 9%**	**24,126,844 ± 22%**	**19%**

**Table 3:** Measurements based on the segmentation of specimens in a sample at 865 cm below the sea floor of core GeoB 8502-2, corresponding to 139.1 Kyrs before present.

Sample 865 cm	Interactive 3D Models	Shell Volume (μm^3^)	Shell Area (μm^2^)	Shell thickness (μm)	Cell Volume (μm^3^)	Dirt
GeoB 8502-2_Depth865_Shell 1	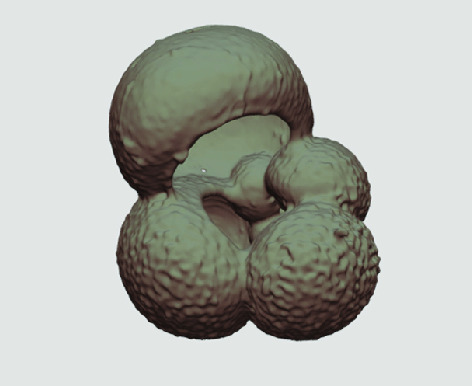	4,171,795	926,927	4.5	19,457,800	2%
GeoB 8502-2_Depth865_Shell 2	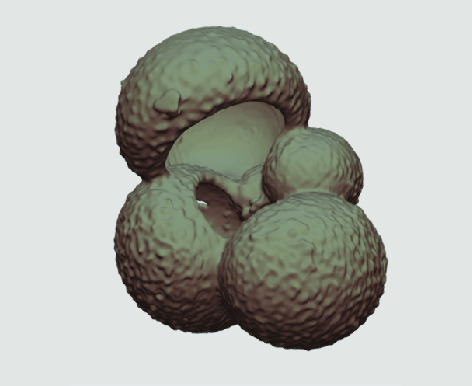	4,570,961	1,058,658	4.3	23,419,300	1%
GeoB 8502-2_Depth865_Shell 3	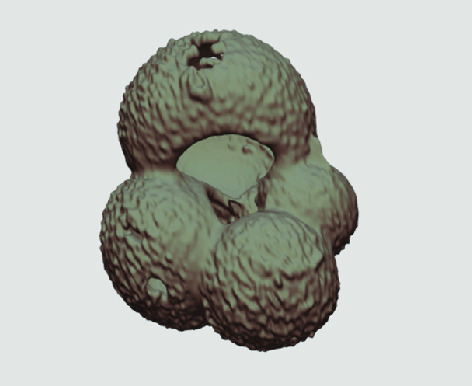	3,401,100	654,190	5.2	11,831,500	5%
GeoB 8502-2_Depth865_Shell 4	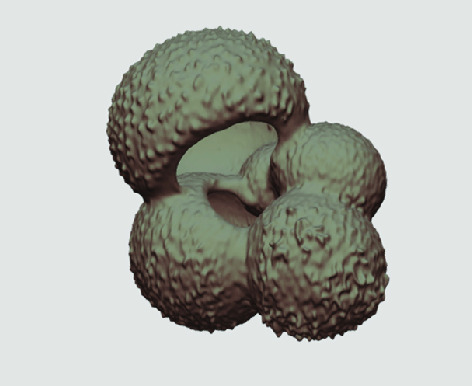	5,002,276	913,557	5.5	18,774,900	0%
GeoB 8502-2_Depth865_Shell 5	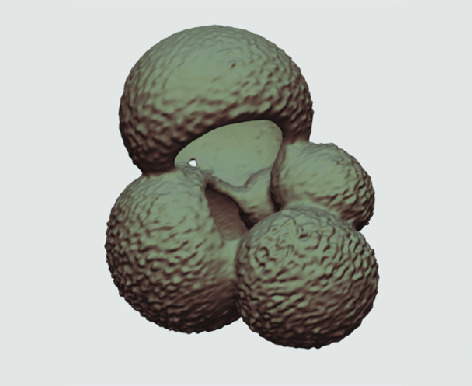	4,173,558	903,174	4.6	18,207,700	5%
GeoB 8502-2_Depth865_Shell 6	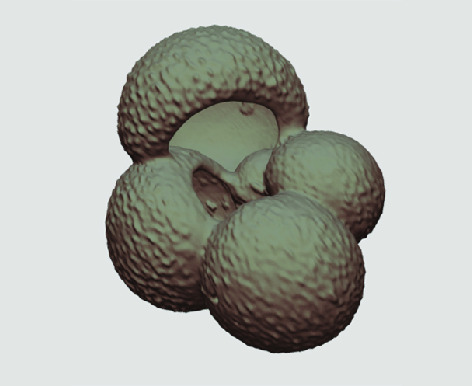	4,052,656	947,705	4.3	19,036,800	4%
GeoB 8502-2_Depth865_Shell 7	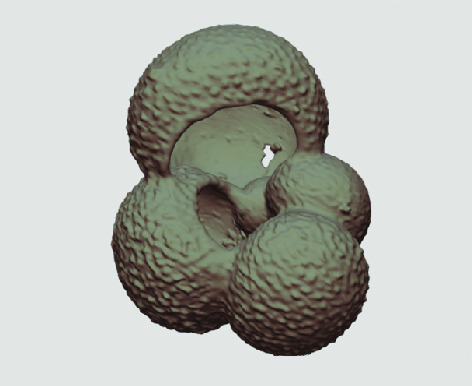	4,917,115	985,332	5.0	21,232,100	9%
GeoB 8502-2_Depth865_Shell 8	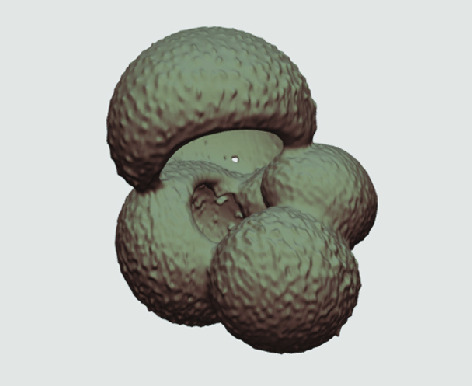	5,439,635	967,690	5.6	21,157,000	6%
GeoB 8502-2_Depth865_Shell 9	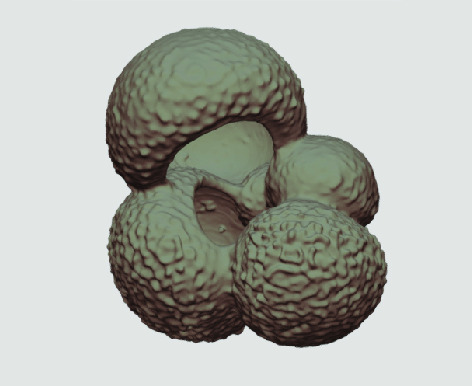	3,847,958	910,403	4.2	18,705,500	7%
**Average**		**4,397,450 ± 15%**	**918,626 ± 12%**	**4.8 ± 11%**	**19,091,400 ± 17%**	**4%**

## Discussion

Acquisition and segmentation of tomographic images is a tedious and time-consuming task. To this extent, the availability of virtual micropaleontological specimens, together with curated CT scan data, is an innovative characteristic of the present work. The provided 3D foraminifera shell volumes in HMASCII format retain the segmentation coordinates and can be used together with the TIFF image stack in any CT image analysis software to mask out the pixel areas that correspond to each specimen. This can be particularly useful for studies that focus on the grayscale intensity distribution of the image stack in the present dataset.

**Figure 4.**
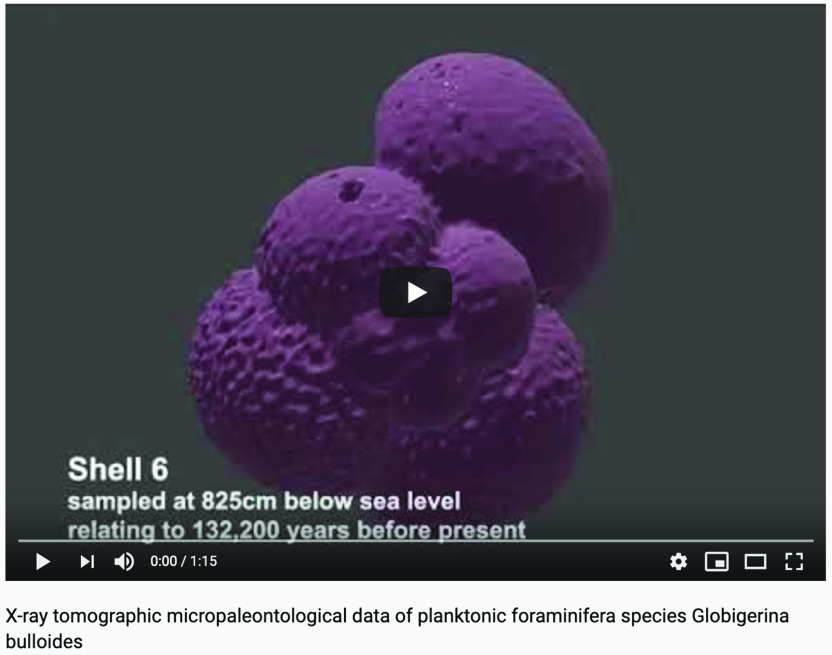
Video of example X-ray tomographic micropaleontological data *from Globigerina bulloides* sampled at three different core depths. See: https://youtu.be/gQ0VoE4YNbc

X-ray microscopy and accessible curated CT data in processable forms can offer vast amounts of morphological data on foraminifera fossils and have the potential to promote better (more accurate) species descriptions (see video summary (Figure 4)). The availability of larger databases can advance virtual and distant learning, or complement computer-aided species digital identification software [15] to conduct larger-scale morphometric studies of foraminifera that can help to quantify the ecological and evolutionary dynamics of taxa in space and time. Apart from studies that uses CT analysis to examine biometrical foraminifera parameters [9, 10, 16], there are studies that can use the pixel’s gray levels to extract geochemical environmental information [6], or information about the corrosion state of shells [17]. Knowledge of the degree of shell dissolution is of key importance in the study of foraminifera shell mass.

CT information is particularly useful in the study of foraminifera shell mass because in order to ascertain the cause behind shell mass changes in the paleoceanographic record, it is important to have foraminifera biometric information (cell volumes) and information about the quantity of calcite mass loss to dissolution [13]. It is also important to know the degree of contamination caused by sediment infilling of chambers, which – as shown in the recent study [4] – can yield considerable artifacts.

## Conclusions

Micro-CT scanning is proving to be a particularly useful tool in micropaleontological research. Apart from providing information about fossil specimen morphometry or state of preservation, it can be also used to determine and quantify the degree of specimen cleanliness.

## Data Availability

Supporting data is available in the *GigaScience* GigaDB repository [18]. Sketchfab specimen 3D models are also available [19], alongside 3D printable models in the Thingiverse repository [20].

## List of Abbreviations

CT: Computed Tomography; Kyrs: kilo (thousand) years
